# V-EHS needs more studies to consolidate its use in clinical practice

**DOI:** 10.1590/S1677-5538.IBJU.2024.9927.1

**Published:** 2025-01-10

**Authors:** Rodrigo Barros

**Affiliations:** 1 Universidade Federal Fluminense Hospital Universitário Antônio Pedro Niterói RJ Brasil Serviço de Urologia, Hospital Universitário Antônio Pedro - Universidade Federal Fluminense - UFF, Niterói, RJ, Brasil

## Comment

The current literature shows that low-intensity extracorporeal shock wave therapy (LI-EWST) appears to be effective and safe for treatment of erectile dysfunction (ED) (1). In this manuscript, the authors report that LI-ESWT is effective for the treatment of moderate vasculogenic ED, with optimal results after 6 months. However, care is needed regarding these findings, especially due to the limitations of the study, including the small sample size and short follow-up. Moreover, since the first publication about LI-EWST by Vardi in 2010, there have been no high-quality studies that allow establishing the patient profile, type of energy, and ideal application protocol needed to achieve clinically satisfactory results (2).

In addition, the study tries to define the best tool for routine clinical assessment of ED using the International Index of Erectile Function (IIEF-5), V-EHS (a new visual scale), and standardized penile Doppler ultrasound before and after LI-EWST. The authors observed a strong correlation between V-EHS and IIEF-5 in the shock wave group. The V-EHS is derived from the original EHS (Erection Hardness Score) (3), but incorporates some modifications. The EHS is a single-item ("How would you rate the hardness of your erection?") patient-reported outcome for scoring erection hardness. Although the EHS is a simple, valid and reliable tool, it is a patient's subjective measurement of his own erection hardness. In V-EHS, the patient does not assign the score subjectively and the erection hardness assessment is made by the examiner. Furthermore, the score is obtained according to the axial resistance that the penis supports after a force applied by the examiner to its tip during a drug-induced erection. Therefore, the V-EHS is not exclusively visual; instead, it is a modified EHS in which the examiner evaluates the axial rigidity mechanically.

Erection hardness is a reflection of axial penile rigidity and characterizes the ability to penetrate and achieve successful intercourse without penile bending (4). Therefore, V-EHS is a very interesting score which is directly related to the penetration capacity and has a strong correlation with the IIEF-5. Thus, it is a valuable tool for clinical practice, especially when an ultrasound device is not available. It can be an adequate substitute for penile Doppler in the evaluation of ED. Despite this, the V-EHS needs more studies with better quality and larger samples to consolidate its use in clinical practice.
